# Transcriptome profiling of five brain regions in a 6‐hydroxydopamine rat model of Parkinson’s disease

**DOI:** 10.1111/cns.13702

**Published:** 2021-08-04

**Authors:** Ying Lyu, Yiying Huang, Guiying Shi, Xuepei Lei, Keya Li, Ran Zhou, Lin Bai, Chuan Qin

**Affiliations:** ^1^ Institute of Laboratory Animal Sciences (ILAS), Chinese Academy of Medical Sciences (CAMS) & Comparative Medical Center Peking Union Medical College (PUMC) Beijing China; ^2^ Beijing Engineering Research Center for Experimental Animal Models of Human Critical Diseases Beijing China; ^3^ Guangzhou Medical University Guangzhou Guangdong China; ^4^ Beijing City University Beijing China

**Keywords:** 6‐hydroxydopamine, different brain regions, Parkinson's disease, synapse, transcriptome

## Abstract

**Background:**

Parkinson's disease (PD) is a neurodegenerative disease, and its pathogenesis is unclear. Previous studies mainly focus on the lesions of substantia nigra (SN) and striatum (Str) in PD. However, lesions are not limited. The olfactory bulb (OB), subventricular zone (SVZ), and hippocampus (Hippo) are also affected in PD.

**Aim:**

To reveal gene expression changes in the five brain regions (OB, SVZ, Str, SN, and Hippo), and to look for potential candidate genes and pathways that may be correlated with the pathogenesis of PD.

**Materials and methods:**

We established control group and 6‐hydroxydopamine (6‐OHDA) PD model group, and detected gene expressions in the five brain regions using RNA‐seq and real‐time quantitative polymerase chain reaction (RT‐qPCR). We further analyzed the RNA‐seq data by bioinformatics.

**Results:**

We identified differentially expressed genes (DEGs) in all five brain regions. The DEGs were significantly enriched in the “dopaminergic synapse” and “retrograde endocannabinoid signaling,” and Gi/o‐GIRK is the shared cascade in the two pathways. We further identified Ephx2, Fam111a, and Gng2 as the potential candidate genes in the pathogenesis of PD for further studies.

**Conclusion:**

Our study suggested that gene expressions change in the five brain regions following exposure to 6‐OHDA. The “dopaminergic synapse,” “retrograde endocannabinoid signaling,” and Gi/o‐GIRK may be the key pathways and cascade of the synaptic damage in 6‐OHDA PD rats. Ephx2, Fam111a, and Gng2 may play critical roles in the pathogenesis of PD.

## Graphical abstract

This study suggested that gene expressions change in the five brain regions following exposure to 6‐OHDA. The “dopaminergic synapse,” “retrograde endocannabinoid signaling,” and Gi/o‐GIRK may be the key pathways and cascade of the synaptic damage in 6‐OHDA PD rats. Ephx2, Fam111a and Gng2 may play critical roles in the pathogenesis of PD.

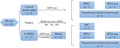

## 1 INTRODUCTION

Parkinson's disease (PD) is the second most common neurodegenerative disease in middle‐aged and elderly people, after Alzheimer's disease (AD).^1^ Death of dopaminergic neurons in the substantia nigra (SN) and decreased levels of dopamine (DA) in the striatum (Str) are the main features of PD.^2^ The clinical manifestations of PD include rigidity, posture instability, bradykinesia, and resting tremors.^3^ Non‐motor symptoms include dysosmia, anxiety, and memory disorders.^4^ Currently, the main treatment used for PD includes levodopa‐based drug therapy and deep brain stimulation surgery. However, negative side effects are observed.^5^, ^6^ Therefore, studying the molecular mechanisms is critical to explore and understand how to prevent and treat PD.

Even though the precise etiology of PD is unknown, it is clear that it is heavily influenced by gene‐environment interactions.^7^ In recent years, many studies implementing transcriptomic techniques, such as RNA‐seq or microarrays, have reported large‐scale changes of gene expression in PD. One study found that there were 1226 differentially expressed genes (DEGs) identified in the SN of PD,^8^ among which 19 DEGs significantly enriched in the metabolic sub‐pathways associated with the pathogenesis of PD.^8^ In a transcriptomic study of blood extracted from PD patients, over 200 DEGs were identified involved in the nucleic acid metabolism, immune response, mitochondria functions, and intracellular transport.^9^ RNA‐seq on cerebrospinal fluid and skin taken from PD patients revealed a large number of DEGs and pathways mainly involved in signal transduction, nuclear regulation, mitochondrial function, immune function, and protein metabolism.^10^, ^11^ The key DEGs can affect the biological functions and development of PD. Therefore, using transcriptome to reveal the potential molecular mechanisms and biomarkers of PD is a hot spot and an important means.

At present, studies about brain regions of PD mainly focus on the SN and striatum, which are the major regions with pathological and biochemical changes, but the lesions may be not limited to these two regions. Five brain regions are closely associated with PD, including olfactory bulb (OB), subventricular zone (SVZ), striatum, SN, and hippocampus (Hippo). After birth, adult neurogenesis is currently thought to be restricted to the subgranular zone of hippocampal dentate gyrus as well as the SVZ along the lateral ventricles.^12^ It has been reported that DA is involved in regulating endogenous neural stem cells in both the subgranular zone and SVZ, while lack of DA may affect neurogenesis, and further influences the regeneration of damaged neurons in PD.^13^ The OB is involved in the development of PD. The volume of the OB in PD patients is significantly smaller than that in healthy individuals.^14^ The dysosmia is a risk factor for the development of PD.^15^ Moreover, some researchers believe that the earliest lesions in PD may occur in the non‐substantia nigra as well as surrounding areas including the OB or enteric nervous systems.^16^ However, the gene expressions in these brain regions have not to be fully documented.

In this study, to determine the genetic profile of the five brain regions above, we examined gene expression using RNA‐seq and analyzed the DEGs in the OB, SVZ, striatum, SN, and Hippo. Then, we interpreted the functions of the DEGs using bioinformatics analyses including Venn analysis, Gene Ontology (GO) analysis, Kyoto Encyclopedia of Genes and Genomes (KEGG) analysis, and protein‐protein interaction (PPI) networks. According to the analyses, we further validated some candidate genes using real‐time quantitative polymerase chain reaction (RT‐qPCR). Further studies with more PD models, especially human PD, are required to find the key candidate genes and molecular mechanisms in PD.

## 2 METHODS

### 2.1 Animals

Twenty‐six male specific pathogen‐free (SPF) Sprague Dawley (SD) rats (Beijing HFK Bioscience), weighing 180–220 g, were used in this study (nine rats in saline control group and 17 rats in 6‐OHDA group). The animals were housed in a facility (humidity: 50 ± 5%; temperature: 24 ± 2°C) under a 12/12 h light/dark cycle with food and water ad libitum. All procedures were approved by the Animal Care and Use Committees of Institute of Laboratory Animal Science of Peking Union Medical College (IACUC: BL18001). All animal experiments were carried out in accordance with the Animal Research: Reporting In Vivo Experiments (ARRIVE) 2.0 guidelines.^17^

### 2.2 Surgical procedures and behavioral test

Rats were anesthetized using 2.5% isoflurane and were fixed in a stereotaxic frame on a heated animal bed. The rats were incised along the midline to expose the anterior fontanelle. The site of medial forebrain bundle (MFB) was located and drilled. 6‐OHDA (contained ascorbic acid as stabilizer; 3 μg/μl; Cat# H116, Sigma‐Aldrich) was injected by microsyringe into the left two points of MFB according to the previous report^18^: tooth bar (TB) = −2.3 mm, anterior‐posterior (AP) = −4.4 mm, lateral (L) = 1.2 mm, dorsoventral (DV) = −7.8 mm, dose = 2.5 µl; TB = +3.4 mm, AP = −4.0 mm, L = 0.8 mm, DV = −8.0 mm, dose = 2 µl. The control group was injected with sterile saline. We kept the injection rate slowly (0.5 µl/min) and left the microsyringe in for 3 min before it was retracted slowly.

All rats (in control and 6‐OHDA group) were tested for apomorphine (APO)‐induced rotation at 4, 5, and 6 weeks after surgery to ensure the number and behavior stability of PD models. The rats were injected intraperitoneally with apomorphine (0.5 mg/kg; Cat# 4393, Sigma‐Aldrich) and were induced to rotate toward the non‐surgical side. If the number of rotations within 30 min was more than 210 (≥7 rpm), the rat was considered as a PD model that was suitable for future experimentation.^19^

### 2.3 Immunohistochemistry (IHC) and transmission electron microscope (TEM)

Rats were sacrificed by intraperitoneal injection of 2% sodium pentobarbital and then perfused intracardially with cold 0.9% NaCl followed by being fixed using 4% paraformaldehyde (PFA). The brains of the rats were carefully removed and were post‐fixed in 4% PFA overnight at 4°C. The fixation method described above was common to IHC (PD rats = 3) and TEM (Control group = 3, PD rats = 3).

In the experiment of IHC, the fixed brains were dehydrated in a gradient of 10%, 20%, and 30% sucrose solution. Continuous coronal sections of brain tissues were performed using a frozen microtome with a thickness of 30 μm. The required sections were collected for staining according to the instructions of the IHC kit (Cat# PV‐6001, ZSGB‐BIO). Brain slices were incubated with 3% hydrogen peroxide (H_2_O_2_) and then were blocked with goat serum. Slices were incubated with a primary antibody (anti‐TH, 1:500, Cat# ab6211, Abcam) overnight at 4°C, and the next day, were washed with phosphate‐buffered saline (PBS). The slices were incubated with horseradish enzyme‐labeled secondary antibody IgG polymer, and DAB solution was applied to visualize immunoreactive cells. After thoroughly washed, the sections were dehydrated with gradient ethanol, cleared with xylene. Sections were imaged by a slice scanning machine (NanoZoomer, Hamamatsu) and analyzed by image J software.

In the TEM experiments, the fixed brains were cut into cubes about 1 mm in size and fixed at 4°C 2.5% glutaraldehyde for 2 h. After washed with phosphate buffer, the samples were fixed in 1% osmic acid (4°C, 2 h). The tissues were dehydrated in a gradient of alcohol, replaced by propylene oxide, embedded in Epon 812, cut into semi‐thin sections, and localized with methylene blue staining. Ultrathin sections (90 nm) were cut and stained by uranyl acetate and lead citrate. TEM (JEM‐1400, JEOL) and its imaging system (CCD camera, Gatan) were used to analyze.

### 2.4 High‐performance liquid chromatography

Striatum tissue was dissected on ice and weighed (Control group = 3, PD rats = 3). The tissues were homogenized using OMNI Bead Ruptor (5.65 m/s, 10 s; OMNI) in solution A (0.4 mol/L HCLO_4_, 10 µl/mg), incubated on ice for 1 h, and centrifuged (13683 *g*, 4°C, 20 min). The supernatants were mixed with solution B (20 mM potassium citrate, 300 mM dipotassium hydrogen phosphate, 2 mM EDTA‐2Na; supernatants:solution B = 2:1 Volume), incubated for 1 h on ice, and centrifuged (13683 *g*, 4°C, and 20 min). The final supernatants were collected to detect the concentration of DA, dihydroxyphenyl acetic acid (DOPAC), and homovanillic acid (HVA) by high‐performance liquid chromatography (HPLC) combined with electrochemical detection (HPLC‐ECD; Waters, USA). HPLC buffer (pH = 4.1) consisted of 50 mM citric acid, 50 mM sodium acetate, 0.1 mM EDTA‐2Na, and 0.5 mM sodium octane sulfonate. The conditions of HPLC included the mobile phase (buffer:methanol = 87.2:12.8 Volume), chromatographic column (150 × 4.6 mm, 5 μm, Waters, USA), voltage (0.6 V), and flow rate (0.8 ml/min). Data were expressed as ng DA/DOPAC/HVA per mg tissue.

### 2.5 RNA isolation, library preparation, and sequencing

Five brain regions (OB, SVZ, Str, SN, and Hippo) were quickly dissected on ice and weighed (Control group = 3, PD rats = 3). RNA was extracted using TRIzol reagent (Cat# 15596018, Invitrogen) according to the manufacturer's instructions. After isolating RNA, its integrity was examined using the Agilent 2100 bioanalyzer (Agilent Technologies); its concentration and purity were detected using a NanoPhotometer spectrophotometer (IMPLEN); and its contamination and degradation were evaluated using 1% agarose gels.

Total RNA was used as input material for the RNA sample preparations, and sequencing libraries were generated using a RNA Library Prep Kit (NEB, USA). The mRNA with poly‐A tails was enriched by Oligo (dT) magnetic beads, then fragmented randomly using divalent cations in NEB Fragmentation Buffer. In the M‐MuLV Reverse Transcriptase system, the first strand of cDNA was synthesized using the fragmented mRNA as a template and random oligonucleotides as primers. The second strand of cDNA was synthesized using dNTPs as raw material, DNA polymerase I, and RNaseH. After purification, double‐stranded cDNA was converted into blunt ends, adenylation of 3′ ends, and ligated to NEBNext Adaptor. The cDNA of 200 bp in length was selected using AMPure XP beads (Beckman Coulter) for PCR amplification. Finally, products were purified and library quality was assessed using the Agilent 2100 bioanalyzer. The index‐coded samples were clustered using TruSeq PE Cluster Kit v3‐cBot‐HS (Illumina). Then, the libraries were sequenced by Illumina (service provided by Novogene).

### 2.6 RNA‐seq data analysis

Raw data (raw reads) obtained by sequencing were presented in the fastq format. In order to ensure the quality and reliability of the data, it was necessary to filter and obtain clean reads by removing low‐quality reads, reads with adapter, and reads with ploy‐N. Meanwhile, Q20, Q30, and GC content were calculated (Illunina Casava 1.8) for clean data, which were used in further analyses. HISAT2 v2.0.5 was used to map clean reads to the reference genome (Rnor_6.0). Cuffdiff (v2.1.1) was used to calculate fragments per kilo‐base of exon per million mapped reads (FPKMs) of genes.

Differentially expressed genes analysis was performed using DESeq2 R (1.16.1) to compare the different test groups. The Benjamin and Hochberg method was used to adjust the *p*value to control for error rate. The *p*adj < 0.05 found by DESeq2 was assigned to DEGs. The *p*adj < 0.05 and |log_2_(fold‐change)| > 1 were used as thresholds for significantly DEGs. The Venn tool (http://bioinformatics.psb.ugent.be/webtools/Venn/) was used to compose Venn diagrams for DEGs. The GOseq R package and KOBAS software were used to analyze the enrichment of DEGs in the database of Gene Ontology (GO) and Kyoto Encyclopedia of Genes and Genomes (KEGG). PPI analysis of DEGs was based on a STRING database that predicts protein‐protein interactions. The network was constructed by extracting a list of target genes from the database using Cytoscape software.

### 2.7 Real‐time quantitative polymerase chain reaction

Total RNA was extracted using TRIzol reagent as described above. The cDNA was synthetized using a reverse transcription kit (Cat# RR047A, Takara) according to the manufacturer's procedure. Primers (Table S1) were designed by the Primer‐BLAST designing tool of NCBI website. RT‐qPCR was performed using SYBR^®^ Premix Ex TaqTM II (Cat# RR820A, Takara) on the StepOnePlus™ Real‐Time PCR System (Applied Biosystems). Gapdh was used for an endogenous housekeeping gene control.

### 2.8 Statistical analysis

Data were presented as mean ± SEM and analyzed using SPSS 16.0 software. Tests of normality (Shapiro‐Wilk) were used for all data distribution assessment. Parametric tests were performed on the normally distributed data. Student's *t* test was used for the comparison between two groups. Paired‐samples *t* test was conducted for the comparison of a rat between two time points. *p* < 0.05 was considered statistically significant.

## 3 RESULTS

### 3.1 6‐OHDA lesioned parkinsonian behavior and pathological changes in rats

**FIGURE 1:**
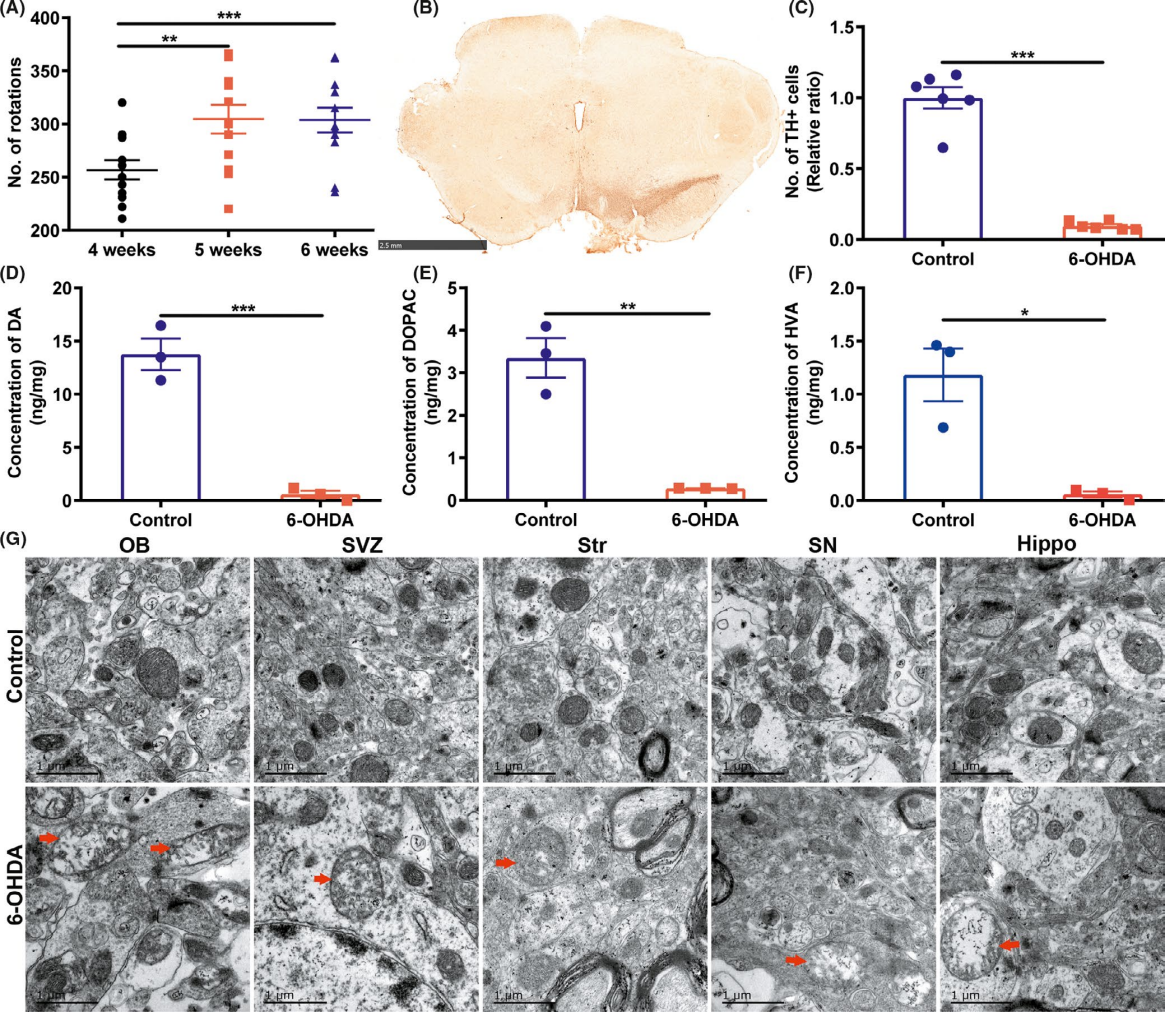
Behavioral test and pathological lesions in rats. (A) The number of rotations induced by APO in PD rats (APO‐induced rotations ≥7 rpm) at the 4, 5, and 6 weeks following exposure to 6‐OHDA (*n* = 12 rats, Paired‐samples *t* test). (B) TH immunohistochemical staining in the SN of 6‐OHDA PD rats (Scale bar = 2.5 mm). (C) The number of TH+ cells in the SN of 6‐OHDA PD rats (*n* = 6 brain slices, Student's *t* test). (D‐F) The concentrations of DA, DOPAC, and HVA in the striatum (*n* = 3 rats per group, Student's *t* test, saline control vs 6‐OHDA PD rats). (G) Ultrastructural changes of mitochondria in five brain regions. Red arrows represent injured mitochondria (saline control vs 6‐OHDA PD rats; Scale bar = 1 μm,). *p < 0.05, **p < 0.01, ***p < 0.001

The APO‐induced rotation test was performed on rats both in the control group and 6‐OHDA group at 4, 5, and 6 weeks after surgery. No rotation behavior was observed in the control group. However, 4 weeks following exposure to 6‐OHDA, part of the rats were induced to rotate toward the non‐surgical side, and 12 out of the 17 rats met the rotation criteria (≥7 rpm, 30 min) and were considered as 6‐OHDA PD models. The number of 6‐OHDA PD rats had no difference at three time points. However, the number of rotations in 5 weeks was more than that in 4 weeks and had no difference compared with that in 6 weeks (Figure 1A), indicated the number of rotations was stable at 6 weeks after surgery.

We stained the dopaminergic neurons in the SN with TH and counted the number of TH+ neurons. The lesion side lost approximately more than 90% TH+ neurons compared with the intact side (Figure 1B,C) in 6‐OHDA PD rats. Then, we detected the concentrations of DA, DOPAC, and HVA in the striatum. Concentrations of DA, DOPAC, and HVA in the lesion side were reduced by more than 90% compared with the control rats (Figure 1D‐F). Furthermore, we observed the ultrastructure of mitochondria in five brain regions. Compared with the control group, the mitochondria of the 6‐OHDA PD model showed varying degrees of degeneration in the five brain regions, containing mitochondria swelled and residual cristae (arrows in Figure 1G).

### 3.2 Differential expression of genes in the five Brain Regions between 6‐OHDA PD models and control rats

**FIGURE 2:**
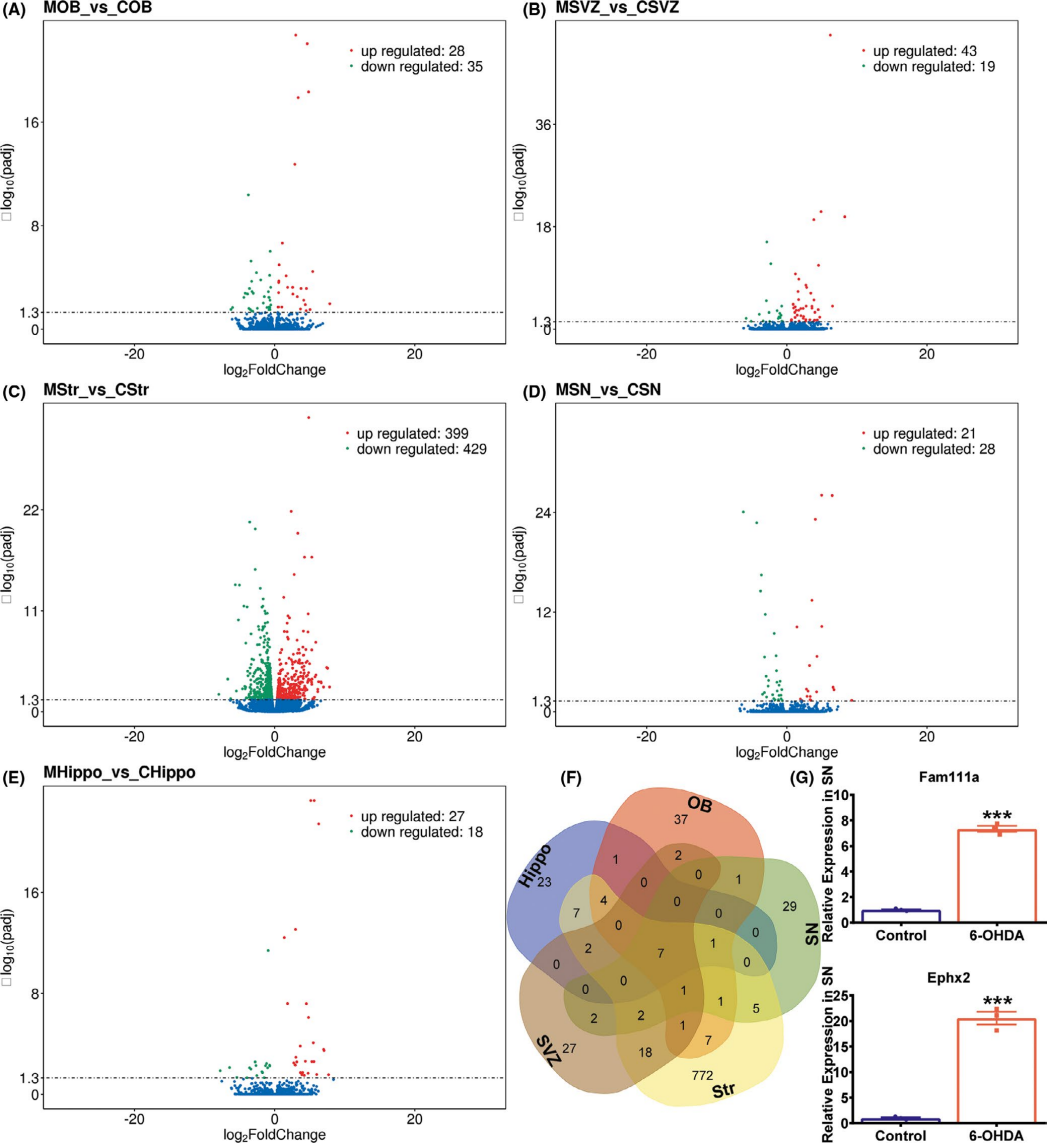
Identification of the DEGs in five brain regions between 6‐OHDA PD rats (*n* = 3 rats) and saline controls (*n* = 3 rats). (A‐E) Volcano plots of the DEGs in the OB, SVZ, Str, SN, and Hippo. The *x*‐axis represents the |log_2_foldchange| of the DEGs. The *y*‐axis represents the statistical significance of the DEGs (*p*adj). Red dots represent the upregulated DEGs, green dots represent the downregulated DEGs, and blue dots represent genes with no significant differences. C = saline control group and M = 6‐OHDA PD rat model. (F) Venn analysis of the DEGs in the five brain regions. (G) Relative mRNA expression of Ephx2 and Fam111a, which were the overlapped DEGs of the five brain regions, were validated using RT‐qPCR in the SN (*n* = 3 rats per group, Student's *t* test, *p < 0.05, **p < 0.01, ***p < 0.001, saline control vs 6‐OHDA PD rats)

To characterize the transcriptomic profiles in the five brain regions of rats exposed to 6‐OHDA, RNA‐Seq was performed with three biological replicates of each group. More than 1446.3 million clean reads were obtained altogether in the library after data filtering and the Q30 quality score for each sample was ≥91.9% (Table S2). 93.17% to 94.04% clean reads mapped to the rat reference genome (Rnor_6.0), and most (87.56%‐88.55%) of them were uniquely mapped (Table S3). FPKM distributions for each sample and the correlation of biological replicates for each group are displayed in Figure S1. The results above indicated that the quality of the RNA‐seq data was credible and suitable for further analysis. We considered *p*adj < 0.05 and |log_2_(fold‐change)| > 1 as thresholds for significantly DEGs. The results showed 63 (28 upregulated and 35 downregulated) DEGs in the OB, 62 (43 upregulated and 19 downregulated) DEGs in the SVZ, 828 (399 upregulated and 429 downregulated) DEGs in the striatum, 49 (21 upregulated and 28 downregulated) DEGs in the SN, and 45 (27 upregulated and 18 downregulated) DEGs in the Hippo (Figure 2A‐E). The top 10 (upregulated and downregulated) DEGs in the five brain regions are presented in Table S4–S8. We further analyzed the overlapped DEGs in the five brain regions by Venn analysis. There were 7 DEGs (Fam111a, Phyhip, Ephx2, Retsat, Anxa6, Rn60_7_1214.2, and LOC100361008) overlapped in all five brain regions (Figure 2F). Among the seven overlapped DEGs, Ephx2 and Fam111a were displayed in the table of top 10 DEGs of the SN (Table S7), and highly expressed in 6‐OHDA PD models (Figure 2G).

### 3.3 GO and KEGG pathway analyses of the DEGs in five brain regions

**FIGURE 3:**
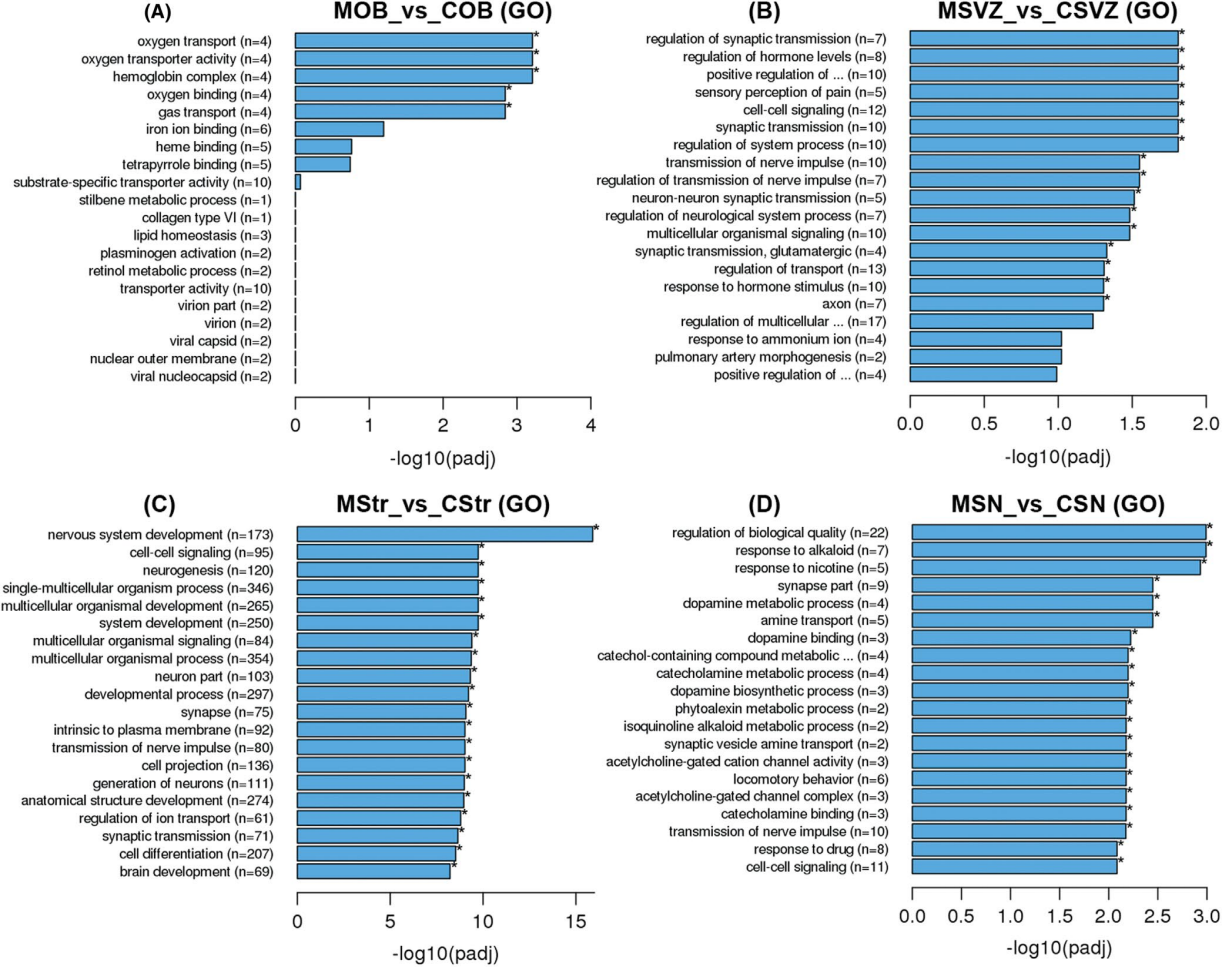
GO analysis of the DEGs in five brain regions between 6‐OHDA PD rats (*n* = 3 rats) and saline controls (*n* = 3 rats). (A‐D) Top 20 GO terms ranked in the OB, SVZ, Str, and SN. The *x*‐axis represents the statistical significance. The *y*‐axis shows the GO terms, (*n*) represents the number of DEGs enriched in this GO term. *padj < 0.05. C = saline control group and M = 6‐OHDA PD rat model

To interpret the biological functions of the DEGs in the five brain regions, we performed GO analysis. In the OB, there were five significantly enriched GO terms, and the DEGs are mainly involved in “oxygen transport,” “oxygen binding,” and “hemoglobin complex” (Figure 3A). In the SVZ, there were 16 significantly enriched GO terms, and the DEGs are mainly involved in “synaptic transmission,” “cell‐cell signaling,” “sensory perception of pain,” “transmission of nerve impulse,” and “regulation of hormone levels” (Figure 3B). In the striatum, a total of 325 significantly enriched GO terms were obtained, and the top 20 GO terms are displayed in Figure 3C mainly associated with “nervous system development,” “neuron part,” “multicellular organismal signaling,” “synapses,” “transmission of nerve impulses,” “generation of neurons,” “regulation of ion transport,” “synaptic transmission,” and “cell differentiation”. In the SN, a total of 46 significantly enriched GO terms were obtained, and the top 20 GO terms are shown in Figure 3D mainly associated with “response to nicotine,” “dopamine metabolic processes,” “catecholamine metabolic processes,” “locomotory behavior,” “acetylcholine‐gated cation channel activity,” “synaptic vesicle amine transport,” and “transmission of nerve impulses”. In the Hippo, no significantly enriched GO term was obtained.

**FIGURE 4:**
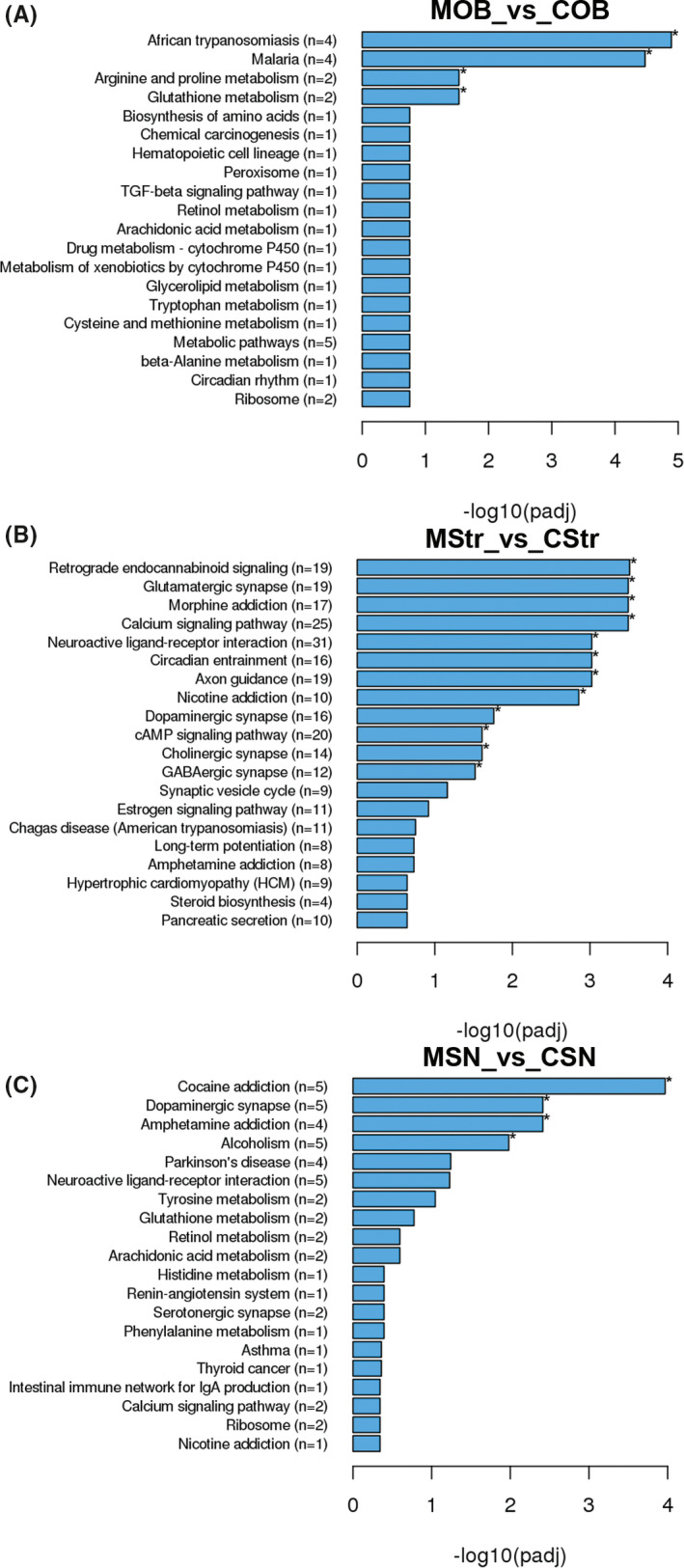
KEGG pathway analysis of the DEGs in five brain regions between 6‐OHDA PD rats (*n* = 3 rats) and saline controls (*n* = 3 rats). (A‐C) Top 20 KEGG terms ranked in the OB, Str and SN. The *x*‐axis represents the statistical significance. The *y*‐axis shows the KEGG terms, (*n*) represents the number of DEGs enriched in this KEGG term. *padj < 0.05. C = saline control group and M = 6‐OHDA PD rat model

**FIGURE 5:**
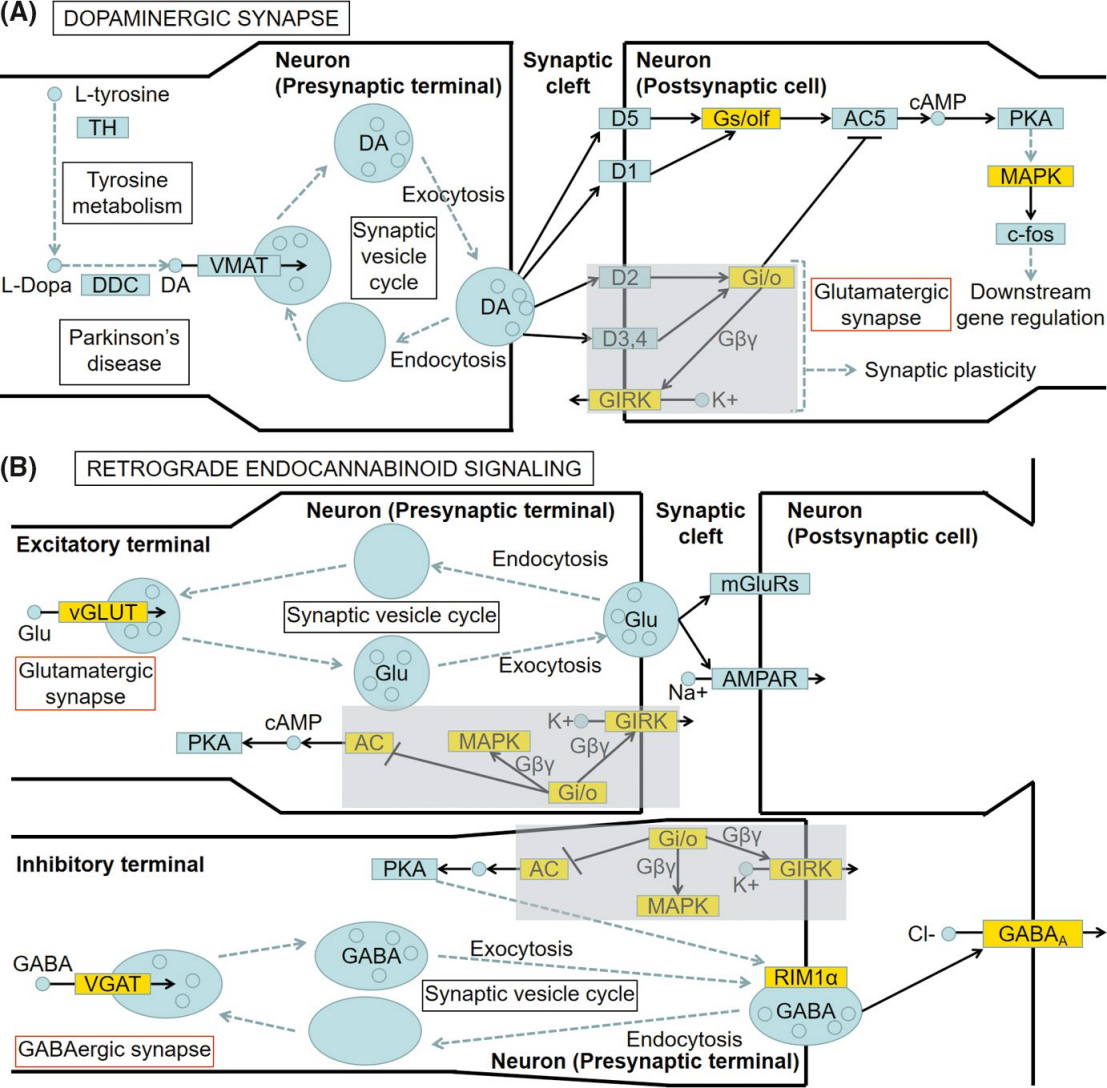
Synopsis of significant “dopaminergic synapse” (A) and “retrograde endocannabinoid signaling” (B) pathways in the striatum of the 6‐OHDA PD rat model according to the KEGG analysis. The yellow blocks represent the enriched DEGs. The gray blocks mark the shared cascade in the two pathways. The red boxes mark the related KEGG pathways. This synopsis referred to the pathways (map04728 Dopaminergic synapse and map04723 Retrograde endocannabinoid signaling) of KEGG (https://www.kegg.jp/kegg/)^45^

**FIGURE 6:**
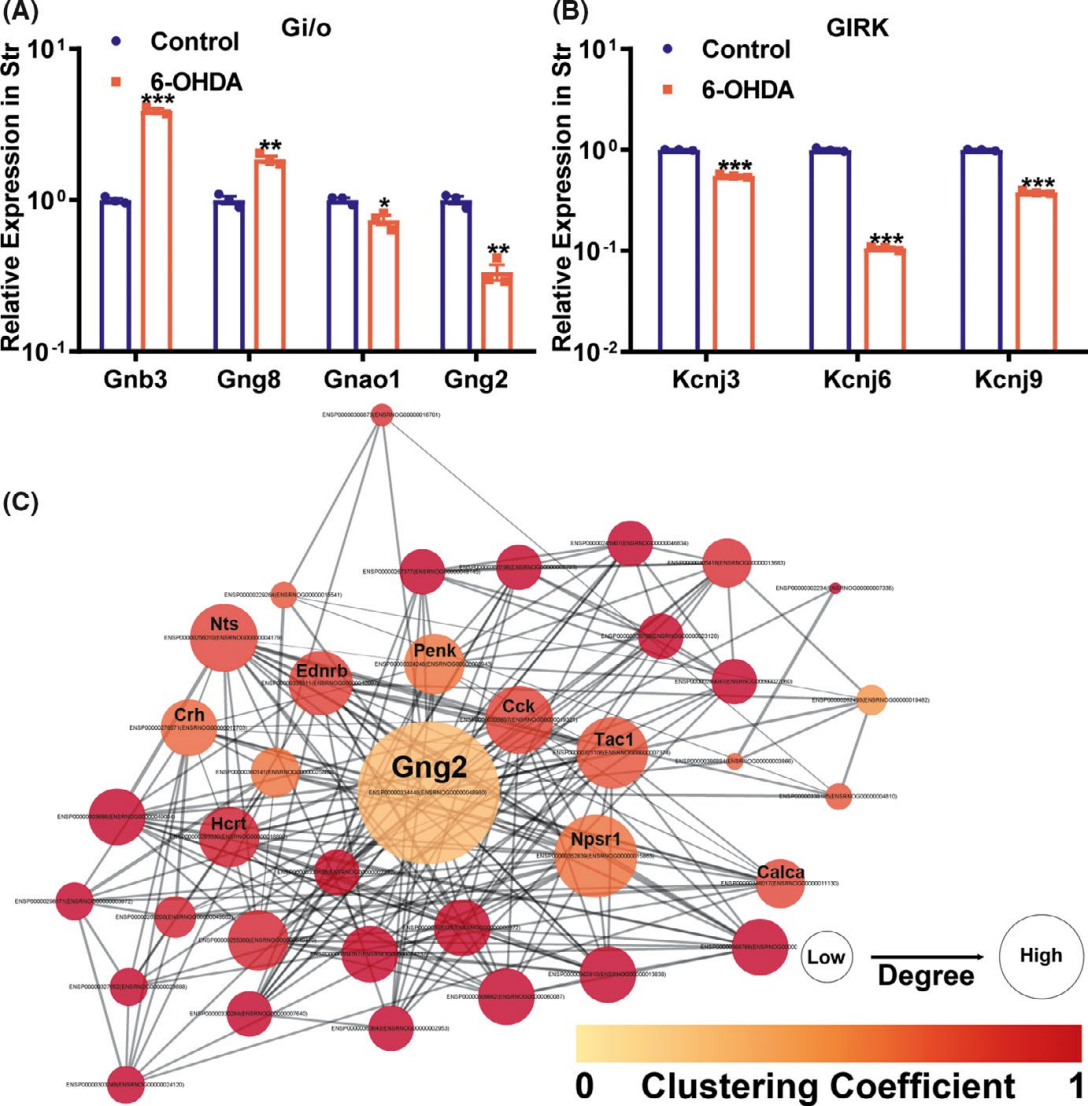
Relative mRNA expression of the DEGs enriched in the Gi/o (A) and GIRK (B) involved in the KEGG pathways of the striatum (*n* = 3 rats per group, Student's *t* test, *p < 0.05, **p < 0.01, ***p < 0.001, saline control vs 6‐OHDA PD rats). (C) The new PPI network of the DEGs enriched in the striatum. The bigger node size represents the bigger degree value. The color bar shows the clustering coefficient

To reveal functional interactions involved in inter‐ and intra‐cellular signaling cascades, we performed KEGG pathway analysis. The significantly enriched KEGG pathways of DEGs in the OB, striatum, and SN regions are displayed in Figure 4. There was no statistically enriched KEGG pathway in the SVZ and Hippo. Remarkably, the “dopaminergic synapse” was the significant KEGG pathway enriched in both the striatum and the SN, and the “retrograde endocannabinoid signaling” was the significant KEGG pathway ranked first in the striatum (Figure 4B,C). Next, we further found the Gi/o‐GIRK is the shared cascade among the DEGs enriched in the two KEGG pathways above in the striatum (Figure 5). In Figure 5, we found the Gi/o‐GIRK is also involved in the “glutamatergic synapse” and “GABAergic synapse” which were significantly enriched in the striatum (Figure 4B). To validate the regulation of the DEGs enriched in the Gi/o (Gnb3/Gng8/Gnao1/Gng2) and GIRK (Kcnj3/Kcnj6/Kcnj9), RT‐qPCR was performed. Gnb3 and Gng8 were highly expressed in the striatum of 6‐OHDA PD models (Figure 6A). Gnao1, Gng2, Kcnj3, Kcnj6, and Kcnj9 were expressed at lower mRNA levels in the striatum of 6‐OHDA PD models compared with the control group (Figure 6). The trends for Gi/o (Gnb3/Gng8/Gnao1/Gng2) and GIRK (Kcnj3/Kcnj6/Kcnj9) validated by RT‐qPCR were consistent with the RNA‐seq results.

### 3.4 PPI analysis of DEGs in five brain regions

To further understand the functional contributions and interactions of the DEGs, PPI network analysis was performed. In the OB and Hippo, the number of high‐degree nodes and the clustering coefficient were generally low (Figure S2A,E). In the SVZ and SN, the number of high‐degree nodes was low, and the clustering coefficient was relatively higher than that in the OB and Hippo (Figure S2B,D). In the striatum, the number of high‐degree nodes and the clustering coefficient were the highest (Figure S2C). The high‐degree nodes with relatively higher clustering coefficient in the striatum were selected to compose a new PPI network (Figure 6C). The new network with a high clustering coefficient (0.848) contained 37 nodes and revealed Gng2 as the most connected node at the center (Figure 6C). Table S9 showed the top 15 node genes ranked by degree in the PPI network of striatum.

## 4 DISCUSSION

In this study, using a transcriptomic approach, we analyzed gene expression in five brain regions from 6‐OHDA PD rats and saline controls. We identified 63 DEGs in the OB, 62 DEGs in the SVZ, 828 DEGs in the striatum, 49 DEGs in the SN, and 45 DEGs in the Hippo. Among the DEGs, Ephx2 and Fam111a were two overlapped genes in the five brain regions. GO and KEGG pathway analyses indicated that the “dopaminergic synapse” and “retrograde endocannabinoid signaling” were modulated in the striatum of 6‐OHDA PD rats. In the two pathways, DEGs were enriched in Gi/o (Gnb3/Gng8/Gnao1/Gng2) and GIRK (Kcnj3/Kcnj6/Kcnj9), and Gi/o‐GIRK is the shared cascade also involved in “glutamatergic synapse” and “GABAergic synapse.” Furthermore, the PPI analysis showed that Gng2 was the center connected with Penk and Crh in the network of striatum (Figure 6C).

The 6‐OHDA rat model is a kind of neurotoxin model of PD. It is widely utilized in the pathogenesis research, drug discovery, cell transplantation, and gene therapy. It has reported that the loss of dopaminergic neurons in the SN and DA deficiency in striatum is the hallmark pathological feature of PD,^20^ and the severity of motor dysfunction is correlated directly with the extent of DA loss and dopaminergic neural death.^21^, ^22^ In our PD rats, 6‐OHDA caused more than 90% loss of dopaminergic neurons in the SN and DA deficiency in striatum, and we performed APO‐induced rotation test three times to ensure the motor dysfunction classic and stable. To control the effect of APO, the APO‐induced rotation test was performed both in 6‐OHDA rats and control rats. It is generally accepted that 6‐OHDA causes mitochondrial dysfunction that is involved in the pathogenesis of PD.^23^ The mitochondrial damage in the striatum of PD models has been reported previously.^24^ In this study, we further found that varying degrees of mitochondrial damage were observed in all five brain regions. Whether the mechanism of mitochondrial damage in different brain regions is similar needs further investigations.

In the results of the RNA‐seq, among the DEGs in the five brain regions (the OB, SVZ, Str, SN, and Hippo), Ephx2 and Fam111a were the overlapped genes identified by Venn analysis. Ephx2 codes soluble epoxide hydrolase (sEH) and plays a key inflammatory role in metabolism of polyunsaturated fatty acids, thus regulating the pathogenesis of neurological diseases.^25^ In the striatum of 1‐methyl‐4‐phenyl‐1,2,3,6‐tetrahydropyridine (MPTP) mice, the expression of sEH is increased, and the genetic knockout of Ephx2 can protect the striatum from the dopaminergic neurotoxicity of MPTP.^25^ Moreover, the expression of sEH is also positively correlated with the ratio of phosphorylated α‐Syn in striatum.^25^ In this study, Ephx2 was increased in 6‐OHDA PD rats, consistent with studies above. Fam111a is FAM111 trypsin‐like peptidase A, and its mutations are associated with hypoparathyroidism and bone development abnormalities.^26^, ^27^ However, its roles in PD have not been reported and need to be clarified.

In this study, GO and KEGG analyses showed that the function and pathway changes enriched by DEGs were mainly concentrated in the striatum and SN of five brain regions. The “dopaminergic synapse” was enriched in both the striatum and SN, and the “retrograde endocannabinoid signaling” was the first KEGG pathway ranked in the striatum. It is generally accepted that degeneration of dopaminergic neurons is the major pathological change in PD,^28^ and the terminals of dopaminergic neurons are thought to be injured prior to neuronal bodies, indicating that PD may arise at the synapse.^29^ Therefore, “dopaminergic synapse” may be a key pathway of PD, and our results of KEGG analysis highlight its significance. The “retrograde endocannabinoid signaling” is a form of neuromodulation by endocannabinoid.^30^ It has reported that the endocannabinoid system regulates the release of neurotransmitters^31^ and synaptic plasticity, especially retrograde control of excitatory or inhibitory synapses.^32^ Moreover, endocannabinoid system is involved in a variety of neurodegenerative diseases including PD^31^ and is an important target for protecting neurons and neurodegeneration. Therefore, to discover the precise molecular cascade and drug targets, gene expression changes following cannabinoid treatment need further study.^33^

In the two pathways, DA and endocannabinoid systems control each other in PD.^34^ The endocannabinoid system alters DA transmission through trans‐synaptic (including GABAergic synapses and glutamatergic synapses) mechanisms^34^ and is involved in the key biological processes of neurodegenerative diseases including glutamate excitotoxicity, excessive excitatory drive, and hypoxia‐ischemia.^35^ In the two pathways, Gi/o‐GIRK is the shared cascade and is also involved in the “glutamatergic synapse” and “GABAergic synapse”, suggesting that Gi/o‐GIRK may be a key molecular mechanism to the regulation of synaptic damage in PD. However, the speculation must be tested further. Related studies report that Gi/o is involved in the activity of neurotransmitter DA.^36^ In Gi/o‐GIRK, GIRK is activated by binding to the G protein‐coupled receptors, specifically responding to Gi/o with high efficiency, precisely regulating neurotransmission and cellular response.^37^ GIRK dysfunction leads to neuronal excitability changes and cell death, and may have significance in the pathophysiology of PD and become a new therapeutic target.^38^ Furthermore, PPI analysis showed that Gng2 was the center node in the new high clustering coefficient network of the striatum. In our study, Gng2 was also one of the DEGs enriched in the Gi/o described above. Gng2 is G protein subunit gamma 2. It has reported that Gng2 can exchange GDP for GTP and subsequently activates downstream effectors^39^, ^40^, ^41^ involved in synaptic transmission.^42^ Whether Gng2 is a key gene involved in the synaptic damage of PD awaits further research.

Moreover, some recent studies using the 6‐OHDA rodent models have suggested several novel potential therapeutic targets for the restoration of dopaminergic neurons and the amelioration of behavior disorders in PD. For example, it has reported that GABAAR^43^ and GluN2D^44^ may be the potential candidates for PD, consistent with the results of changes in the dopaminergic, GABAergic, and glutamatergic synapses in this study. These findings demonstrate that PD is a comprehensive disease requiring multi‐targets combined therapy, and finding the main sticking point is the key to select the therapeutic target. It must be noted that our subjects were 6‐OHDA rat models of PD, so it is not known whether our results are applicable to other animal models and human PD. Further studies with a larger sample size and varied PD models, especially human PD, are required to discover the major contributed genes and molecular mechanisms in PD.

In conclusion, we discovered that exposure to 6‐OHDA affects gene expression in the OB, SVZ, striatum, SN, and Hippo. The results provided a comprehensive analysis of the DEGs in the five brain regions and indicated significant roles of “dopaminergic synapse” and “retrograde endocannabinoid signaling,” and further led us to speculate Gi/o‐GIRK may be a key molecular cascade of the synaptic damage in our rats with PD. Moreover, Ephx2, Fam111a, Gng2, Gnb3, Gng8, Gnao1, Kcnj3, Kcnj6, and Kcnj9 may play critical roles in the pathogenesis of PD, providing a foundation for further studies into the molecular mechanisms of PD.

## CONFLICT OF INTEREST

None.

## AUTHOR CONTRIBUTIONS

Chuan Qin and Lin Bai designed the experiment and reviewed the article. Ying Lyu, Yiying Huang, Xuepei Lei, Keya Li, and Ran Zhou performed animal experiments and acquired data. Ying Lyu and Guiying Shi performed molecular biology experiments and acquired data. Ying Lyu wrote the article. All authors read and approved the final manuscript.

## Supporting information

Figure S1

Figure S2

Tables S1‐S9

## Data Availability

The data used to support the findings of this study are available from the corresponding author upon request. The raw data of RNA‐seq has been uploaded to the National Center for Biotechnology Information (NCBI, http://www.ncbi.nlm.nih.gov/bioproject/742511, BioProject ID: PRJNA742511).
