# Supplementary figures and images for: Transcriptome profiling of five brain regions in a 6‐hydroxydopamine rat model of Parkinson’s disease
Source: CNS Neurosci Ther. 2021 Aug 4;27(11):1289–99. doi: 10.1111/cns.13702 (PMC8504527; doi:10.1111/cns.13702)

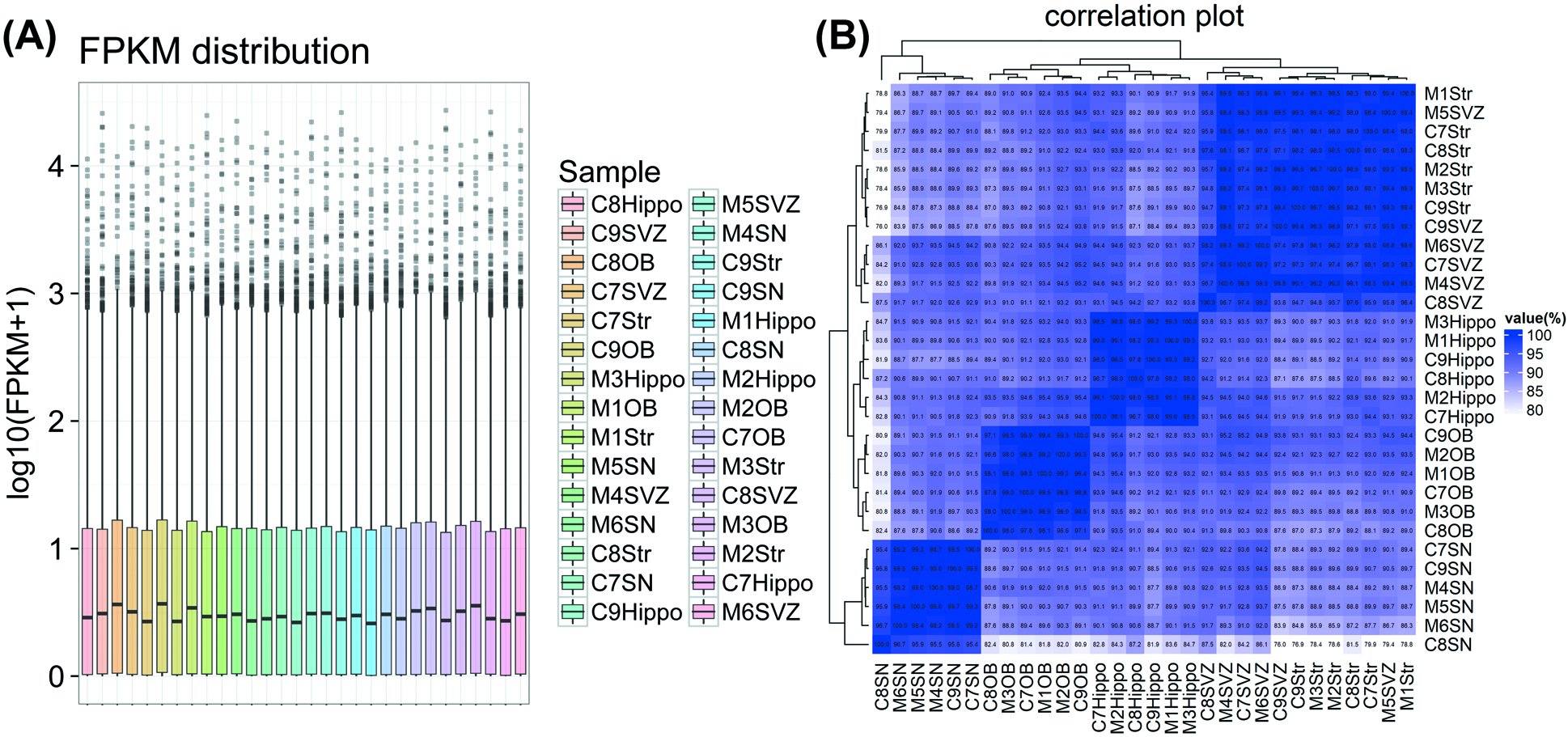

Supplement: Supplementary file 1 — Figure S1 [file CNS-27-1289-s001.tif]

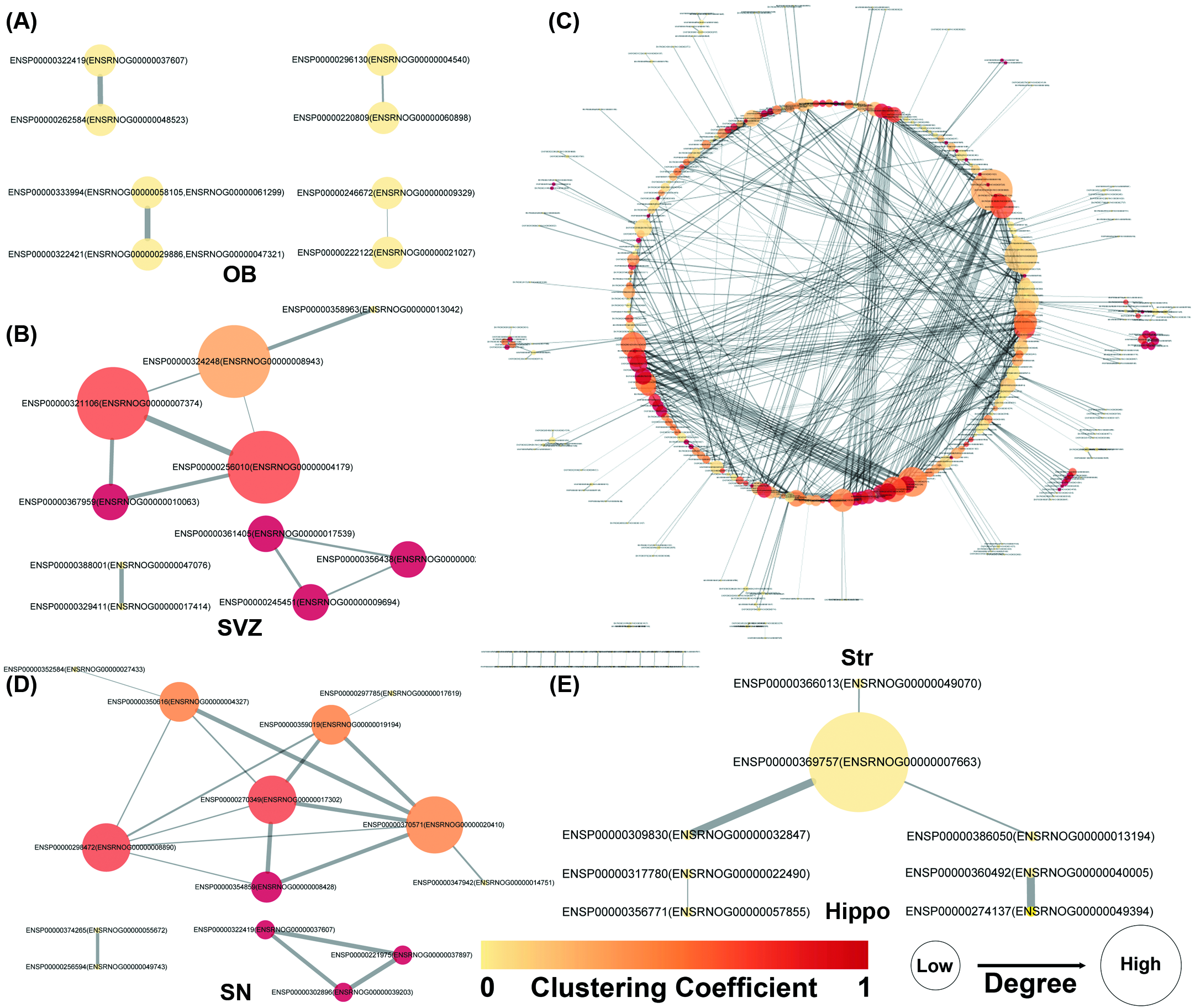

Supplement: Supplementary file 2 — Figure S2 [file CNS-27-1289-s003.tif]
