# Supplementary material for: Transcriptome profiling of five brain regions in a 6‐hydroxydopamine rat model of Parkinson’s disease
Source: CNS Neurosci Ther. 2021 Aug 4;27(11):1289–99. doi: 10.1111/cns.13702 (PMC8504527; doi:10.1111/cns.13702)
Supplement: Supplementary file 3 — Tables S1‐S9 [file CNS-27-1289-s002.doc]

**Supplementary Table S1.** List of primers used for RT-qPCR.

| Genes | Forward primer | Reverse primer |
| --- | --- | --- |
| Gapdh | AGTGCCAGCCTCGTCTCATA | GATGGTGATGGGTTTCCCGT |
| Fam111a | GTGTGGAAAGATCTGGTTGAGGA | GGATCTTCTGTGATTTGCGCT |
| Ephx2 | GCTGCCCAGAGACTTCCTACT | TCCAGCCAGTTGTTGGTGAC |
| Gnb3 | CGTCCGTAGCCTTCTCACTC | AAAGAACGCCTACACGCTCA |
| Gng8 | TCGATCGCATGAAGGTGTCG | CCGTGCCTCATTTTCCCCTA |
| Gnao1 | TGAATACCCAGGCTCCAACAC | ACAAAGGCCAAAGGTCATGCT |
| Gng2 | CGGAGCCAAGCAAGTCTGAT | AGTTTCCTGGCTTGCGCTAT |
| Kcnj3 | GCAAGCTGCTCAAATCTCGG | CATCCCTGTGGTTTCCACGA |
| Kcnj6 | CGGGGCTGATGTGAAATTGG | TTGTGCTTTTCCTTGTGGTGGA |
| Kcnj9 | TTGGGGGAGATGGTGTCAGG | ATCCTGCGGGGTCTGGTTTTG |

**Supplementary Table S2.** Summary of the data quality from RNA-seq.

| Sample name | Raw reads | Clean reads | Raw bases(G) | Clean bases(G) | Error rate(%) | Q20(%) | Q30(%) | GC content(%) |
| --- | --- | --- | --- | --- | --- | --- | --- | --- |
| C7OB | 48351430 | 47124698 | 7.25 | 7.07 | 0.03 | 97.61 | 93.47 | 52.53 |
| C8OB | 48085064 | 46703924 | 7.21 | 7.01 | 0.03 | 97.4 | 93.15 | 51.83 |
| C9OB | 54720492 | 53193942 | 8.21 | 7.98 | 0.03 | 97.36 | 93 | 51.63 |
| M1OB | 48255142 | 46997152 | 7.24 | 7.05 | 0.03 | 97.69 | 93.68 | 52.11 |
| M2OB | 40682442 | 39469484 | 6.1 | 5.92 | 0.03 | 97.9 | 94.18 | 52.4 |
| M3OB | 48298388 | 46992258 | 7.24 | 7.05 | 0.03 | 97.91 | 94.19 | 52.41 |
| C7SVZ | 45273448 | 43749020 | 6.79 | 6.56 | 0.02 | 98.12 | 94.76 | 52.14 |
| C8SVZ | 46055650 | 44651780 | 6.91 | 6.7 | 0.02 | 98.16 | 94.89 | 52.73 |
| C9SVZ | 59006468 | 57282228 | 8.85 | 8.59 | 0.03 | 96.89 | 91.99 | 51.25 |
| M4SVZ | 60596536 | 58448116 | 9.09 | 8.77 | 0.03 | 97.14 | 92.53 | 52.11 |
| M5SVZ | 60014240 | 58802172 | 9 | 8.82 | 0.03 | 97.14 | 92.52 | 51.41 |
| M6SVZ | 49288260 | 48116300 | 7.39 | 7.22 | 0.03 | 97.87 | 94.07 | 52.11 |
| C7Str | 51156602 | 49613012 | 7.67 | 7.44 | 0.03 | 96.86 | 91.94 | 52.07 |
| C8Str | 45993592 | 44507062 | 6.9 | 6.68 | 0.03 | 97.25 | 92.8 | 51.99 |
| C9Str | 45727606 | 44415280 | 6.86 | 6.66 | 0.03 | 97.29 | 92.85 | 51.9 |
| M1Str | 48575208 | 47201960 | 7.29 | 7.08 | 0.02 | 97.99 | 94.42 | 52.29 |
| M2Str | 52527910 | 50930978 | 7.88 | 7.64 | 0.03 | 97.06 | 92.35 | 51.45 |
| M3Str | 57467650 | 55603576 | 8.62 | 8.34 | 0.03 | 97.24 | 92.72 | 51.37 |
| C7SN | 46597816 | 45336314 | 6.99 | 6.8 | 0.03 | 97.92 | 94.25 | 52.7 |
| C8SN | 45544918 | 44048192 | 6.83 | 6.61 | 0.02 | 98.27 | 95.18 | 52.58 |
| C9SN | 50030254 | 48599418 | 7.5 | 7.29 | 0.02 | 98.15 | 94.75 | 52.3 |
| M4SN | 46823566 | 45398478 | 7.02 | 6.81 | 0.02 | 98.09 | 94.68 | 52.43 |
| M5SN | 51834988 | 50352620 | 7.78 | 7.55 | 0.03 | 97.74 | 93.77 | 52.05 |
| M6SN | 50395178 | 48936468 | 7.56 | 7.34 | 0.03 | 97.96 | 94.36 | 53.18 |
| C7Hippo | 42623026 | 41634368 | 6.39 | 6.25 | 0.03 | 97.81 | 93.91 | 52.7 |
| C8Hippo | 51163702 | 49728586 | 7.67 | 7.46 | 0.03 | 97.7 | 93.65 | 52.87 |
| C9Hippo | 53555242 | 52088662 | 8.03 | 7.81 | 0.02 | 98.01 | 94.49 | 53.48 |
| M1Hippo | 48348906 | 46176136 | 7.25 | 6.93 | 0.02 | 98.12 | 94.73 | 52.83 |
| M2Hippo | 45449758 | 44196366 | 6.82 | 6.63 | 0.03 | 97.57 | 93.35 | 52.75 |
| M3Hippo | 47392140 | 46035708 | 7.11 | 6.91 | 0.03 | 97.93 | 94.27 | 52.99 |

Note: OB & Str & Hippo (C7, C8, C9, M1, M2, M3); SVZ & SN (C7, C8, C9, M4, M5, M6). C = saline control group, M = 6-OHDA PD rat model.

**Supplementary Table S3.** Overview of the mapped reads.

| Sample name | Total reads | Total mapped | Multiple mapped | Uniquely mapped |
| --- | --- | --- | --- | --- |
| C7OB | 47124698 | 44271922 (93.95%) | 2544050 (5.4%) | 41727872 (88.55%) |
| C8OB | 46703924 | 43729541 (93.63%) | 2530602 (5.42%) | 41198939 (88.21%) |
| C9OB | 53193942 | 49830161 (93.68%) | 2822069 (5.31%) | 47008092 (88.37%) |
| M1OB | 46997152 | 44090704 (93.82%) | 2496557 (5.31%) | 41594147 (88.5%) |
| M2OB | 39469484 | 37021722 (93.8%) | 2127564 (5.39%) | 34894158 (88.41%) |
| M3OB | 46992258 | 44117161 (93.88%) | 2510284 (5.34%) | 41606877 (88.54%) |
| C7SVZ | 43749020 | 41047349 (93.82%) | 2498287 (5.71%) | 38549062 (88.11%) |
| C8SVZ | 44651780 | 41744672 (93.49%) | 2487607 (5.57%) | 39257065 (87.92%) |
| C9SVZ | 57282228 | 53448114 (93.31%) | 3293014 (5.75%) | 50155100 (87.56%) |
| M4SVZ | 58448116 | 54618561 (93.45%) | 3340588 (5.72%) | 51277973 (87.73%) |
| M5SVZ | 58802172 | 55201755 (93.88%) | 3301276 (5.61%) | 51900479 (88.26%) |
| M6SVZ | 48116300 | 45232297 (94.01%) | 2699777 (5.61%) | 42532520 (88.4%) |
| C7Str | 49613012 | 46225599 (93.17%) | 2750024 (5.54%) | 43475575 (87.63%) |
| C8Str | 44507062 | 41620683 (93.51%) | 2505477 (5.63%) | 39115206 (87.89%) |
| C9Str | 44415280 | 41663043 (93.8%) | 2505111 (5.64%) | 39157932 (88.16%) |
| M1Str | 47201960 | 44279697 (93.81%) | 2754400 (5.84%) | 41525297 (87.97%) |
| M2Str | 50930978 | 47665189 (93.59%) | 2910304 (5.71%) | 44754885 (87.87%) |
| M3Str | 55603576 | 52087040 (93.68%) | 3189855 (5.74%) | 48897185 (87.94%) |
| C7SN | 45336314 | 42555042 (93.87%) | 2595516 (5.73%) | 39959526 (88.14%) |
| C8SN | 44048192 | 41309477 (93.78%) | 2532757 (5.75%) | 38776720 (88.03%) |
| C9SN | 48599418 | 45703671 (94.04%) | 2818541 (5.8%) | 42885130 (88.24%) |
| M4SN | 45398478 | 42615127 (93.87%) | 2651220 (5.84%) | 39963907 (88.03%) |
| M5SN | 50352620 | 47194313 (93.73%) | 2901114 (5.76%) | 44293199 (87.97%) |
| M6SN | 48936468 | 45750980 (93.49%) | 2785544 (5.69%) | 42965436 (87.8%) |
| C7Hippo | 41634368 | 39099601 (93.91%) | 2344332 (5.63%) | 36755269 (88.28%) |
| C8Hippo | 49728586 | 46497088 (93.5%) | 2766609 (5.56%) | 43730479 (87.94%) |
| C9Hippo | 52088662 | 48692843 (93.48%) | 2882431 (5.53%) | 45810412 (87.95%) |
| M1Hippo | 46176136 | 43157181 (93.46%) | 2494648 (5.4%) | 40662533 (88.06%) |
| M2Hippo | 44196366 | 41456651 (93.8%) | 2471048 (5.59%) | 38985603 (88.21%) |
| M3Hippo | 46035708 | 42963409 (93.33%) | 2573971 (5.59%) | 40389438 (87.74%) |

Note: OB & Str & Hippo (C7, C8, C9, M1, M2, M3); SVZ & SN (C7, C8, C9, M4, M5, M6). C = saline control group, M = 6-OHDA PD rat model.

**Supplementary Table S4.** The top 10 (upregulated and downregulated) DEGs in the OB between 6-OHDA PD rat models and saline controls.

| Gene_id | Gene_name | COB_FPKM | MOB_FPKM | Log2FoldChange (M/C) | Pvalue | Padj |
| --- | --- | --- | --- | --- | --- | --- |
| ENSRNOG00000051360 | RGD1563270 | 0 | 1.060072168 | 7.869445947 | 2.11E-05 | 0.010668851 |
| ENSRNOG00000025810 | LOC100911713 | 0.898298724 | 38.40906999 | 5.434599206 | 1.83E-08 | 3.44E-05 |
| ENSRNOG00000009735 | Fibcd1 | 0.018531787 | 0.636096652 | 5.049732374 | 8.51E-05 | 0.030420327 |
| ENSRNOG00000061943 | Rn60_7_1214.2 | 0.535325008 | 15.62080892 | 4.846071272 | 6.72E-23 | 4.64E-19 |
| ENSRNOG00000022490 | LOC100361008 | 1.401968713 | 34.93650467 | 4.650911718 | 8.33E-27 | 8.64E-23 |
| ENSRNOG00000053109 | Mrpl53 | 2.290875692 | 52.52612521 | 4.538901376 | 8.14E-07 | 0.000719937 |
| ENSRNOG00000025216 | Alkbh6 | 0.16390989 | 3.791761412 | 4.50065412 | 0.000109801 | 0.03733584 |
| ENSRNOG00000012067 | Fam111a | 0.105854279 | 2.177485572 | 4.375338344 | 2.59E-05 | 0.012780028 |
| ENSRNOG00000049878 | Sytl2 | 0.085867066 | 1.564535392 | 4.153896211 | 1.07E-05 | 0.005759679 |
| ENSRNOG00000046379 | LOC100912604 | 0.835014259 | 11.26660702 | 3.765895378 | 8.68E-07 | 0.000719937 |
| ENSRNOG00000037607 | LOC100911575 | 108.8635656 | 10.23748727 | -3.372000811 | 2.33E-09 | 5.38E-06 |
| ENSRNOG00000036674 | Cd7 | 3.244906013 | 0.277364241 | -3.49789323 | 7.89E-05 | 0.029226051 |
| ENSRNOG00000016857 | Zg16 | 1.291307153 | 0.098152978 | -3.592926477 | 8.65E-07 | 0.000719937 |
| ENSRNOG00000060898 | LOC100910418 | 6.983517092 | 0.532465761 | -3.675599949 | 6.41E-05 | 0.024632165 |
| ENSRNOG00000020025 | Slc29a2 | 3.224103924 | 0.235614543 | -3.747459689 | 1.20E-14 | 4.14E-11 |
| ENSRNOG00000018148 | Cd99l2 | 1.531682885 | 0.101206027 | -3.862529717 | 2.84E-06 | 0.001903279 |
| ENSRNOG00000027433 | LOC100910708 | 1.165956622 | 0.058216788 | -4.193441547 | 2.33E-06 | 0.001652391 |
| ENSRNOG00000060990 | AABR07000382.1 | 3.385144416 | 0.155528411 | -4.388731369 | 6.15E-06 | 0.00350366 |
| ENSRNOG00000024630 | Igsf23 | 0.338095593 | 0 | -6.00500012 | 5.03E-05 | 0.021389459 |
| ENSRNOG00000006579 | Reg3g | 0.361441449 | 0 | -6.231342752 | 8.16E-05 | 0.02969528 |

Note: C = saline control group (n = 3 rats), M = 6-OHDA PD rat model (n = 3 rats).

**Supplementary Table S5.** The top 10 (upregulated and downregulated) DEGs in the SVZ between 6-OHDA PD rat models and saline controls.

| Gene_id | Gene_name | CSVZ_FPKM | MSVZ_FPKM | Log2FoldChange (M/C) | Pvalue | Padj |
| --- | --- | --- | --- | --- | --- | --- |
| ENSRNOG00000052828 | AABR07017693.1 | 15.35316739 | 12.07227109 | 8.263310927 | 2.59E-24 | 1.77E-20 |
| ENSRNOG00000061943 | Rn60_7_1214.2 | 11.53863429 | 13.79114325 | 6.515935555 | 8.25E-08 | 8.47E-05 |
| ENSRNOG00000004247 | Nhp2 | COB_FPKM | 38.95936486 | 6.204463362 | 9.93E-57 | 2.04E-52 |
| ENSRNOG00000022490 | LOC100361008 | 1.029477374 | 30.62796921 | 4.88108164 | 2.23E-25 | 2.29E-21 |
| ENSRNOG00000028417 | Neurod2 | 0.180224291 | 4.589104828 | 4.691346498 | 1.47E-05 | 0.007340109 |
| ENSRNOG00000012067 | Fam111a | 0.263200105 | 5.916307249 | 4.519848704 | 2.09E-15 | 6.13E-12 |
| ENSRNOG00000016164 | Fcrl2 | 0.209158883 | 4.650784311 | 4.471693937 | 4.86E-07 | 0.000369945 |
| ENSRNOG00000020650 | Slc17a7 | 2.385864622 | 46.90511296 | 4.335915719 | 5.72E-05 | 0.023471208 |
| ENSRNOG00000021513 | Rtn4rl2 | 0.262783662 | 4.143177592 | 4.000184425 | 1.62E-06 | 0.001071372 |
| ENSRNOG00000047642 | AABR07051426.1 | 0.034199642 | 0.560560897 | 3.951549132 | 6.39E-05 | 0.025715009 |
| ENSRNOG00000008312 | Stra6 | 7.278562171 | 2.747038525 | -1.391422653 | 8.18E-07 | 0.000559806 |
| ENSRNOG00000005659 | Aurkb | 16.10518203 | 3.239733986 | -2.287861523 | 8.79E-16 | 3.01E-12 |
| ENSRNOG00000012557 | Lgals5 | 1.414378024 | 0.246822519 | -2.448147504 | 0.000127011 | 0.043594915 |
| ENSRNOG00000017414 | Irf7 | 10.41476194 | 1.787533679 | -2.544616381 | 1.88E-06 | 0.001168035 |
| ENSRNOG00000048016 | LOC102556337 | 24.43039086 | 3.326817266 | -2.859405766 | 1.13E-19 | 4.65E-16 |
| ENSRNOG00000048231 | Tmem235 | 5.274595118 | 0.693229337 | -2.920250141 | 7.54E-09 | 9.68E-06 |
| ENSRNOG00000047076 | Oas1g | 0.40151647 | 0.037485563 | -3.381905313 | 9.50E-05 | 0.036792646 |
| ENSRNOG00000048561 | Hprt1 | 10.36811688 | 0.654405774 | -3.934906535 | 3.93E-06 | 0.002304255 |
| ENSRNOG00000052226 | Ndufa1 | 15.35316739 | 0.44145116 | -5.098849717 | 0.000115097 | 0.040748235 |
| ENSRNOG00000011207 | Gh1 | 11.53863429 | 0.197454597 | -5.814468784 | 2.81E-05 | 0.012803649 |

Note: C = saline control group (n = 3 rats), M = 6-OHDA PD rat model (n = 3 rats).

**Supplementary Table S6.** The top 10 (upregulated and downregulated) DEGs in the striatum between 6-OHDA PD rat models and saline controls.

| Gene_id | Gene_name | CStr_FPKM | MStr_FPKM | Log2FoldChange (M/C) | Pvalue | Padj |
| --- | --- | --- | --- | --- | --- | --- |
| ENSRNOG00000060523 | Rps2-ps2 | 5.436951876 | 0.016743989 | 7.964481898 | 0.000321759 | 0.012435632 |
| ENSRNOG00000007335 | Ccl11 | 0.372103668 | 0 | 6.687787337 | 2.69E-06 | 0.0002811 |
| ENSRNOG00000026051 | RGD1562652 | 2.184401491 | 0.026024869 | 6.273517181 | 0.001347019 | 0.037603546 |
| ENSRNOG00000019301 | Lipm | 1.469990802 | 0.028621559 | 5.611720091 | 7.27E-18 | 1.48E-14 |
| ENSRNOG00000020009 | Npas4 | 11.16858132 | 0.250472918 | 5.463862204 | 0.000113829 | 0.00553334 |
| ENSRNOG00000003959 | Rgs18 | 2.530980611 | 0.060086401 | 5.174940813 | 1.22E-13 | 9.92E-11 |
| ENSRNOG00000006776 | Smyd1 | 1.763596938 | 0.052622088 | 5.019474865 | 8.52E-18 | 1.58E-14 |
| ENSRNOG00000052368 | Clcnka | 0.127111072 | 0.004073599 | 4.590716159 | 5.30E-05 | 0.003139888 |
| ENSRNOG00000037687 | Rspo2 | 3.962720293 | 0.188588798 | 4.387887447 | 2.28E-15 | 3.10E-12 |
| ENSRNOG00000048321 | Tnfsf8 | 0.106442265 | 0.004467967 | 4.262148952 | 2.43E-05 | 0.001685489 |
| ENSRNOG00000059519 | Bbox1 | 0.005589326 | 0.485797582 | -5.896028166 | 7.88E-07 | 9.97E-05 |
| ENSRNOG00000031805 | Krt36 | 0 | 0.183140918 | -6.154759262 | 6.10E-05 | 0.003469621 |
| ENSRNOG00000048232 | Olr1747-ps | 0 | 0.269086123 | -6.155727246 | 0.000135138 | 0.006371518 |
| ENSRNOG00000016953 | Chst4 | 0 | 0.123935701 | -6.270637717 | 6.01E-06 | 0.000534746 |
| ENSRNOG00000005947 | Pde6h | 0 | 0.763722278 | -6.607509847 | 6.30E-06 | 0.000555527 |
| ENSRNOG00000054900 | Pih1d3 | 0 | 0.258679624 | -6.649368187 | 8.89E-06 | 0.000727334 |
| ENSRNOG00000012618 | Efhb | 0 | 0.150831699 | -6.975781098 | 3.00E-05 | 0.002018918 |
| ENSRNOG00000060344 | LOC100911337 | 0 | 1.319133078 | -7.427853029 | 8.10E-08 | 1.49E-05 |
| ENSRNOG00000008807 | Rp1 | 0 | 0.098154218 | -7.556798428 | 1.07E-07 | 1.80E-05 |
| ENSRNOG00000051360 | RGD1563270 | 0 | 1.007504795 | -7.83629289 | 2.98E-05 | 0.002012505 |

Note: C = saline control group (n = 3 rats), M = 6-OHDA PD rat model (n = 3 rats).

**Supplementary Table S7.** The top 10 (upregulated and downregulated) DEGs in the SN between 6-OHDA PD rat models and saline controls.

| Gene_id | Gene_name | CSN_FPKM | MSN_FPKM | Log2FoldChange (M/C) | Pvalue | Padj |
| --- | --- | --- | --- | --- | --- | --- |
| ENSRNOG00000052828 | AABR07017693.1 | 0 | 0 | 11.28478001 | 1.79E-40 | 3.62E-36 |
| ENSRNOG00000049123 | AABR07066510.1 | 0 | 0 | 9.258290304 | 9.59E-05 | 0.041283854 |
| ENSRNOG00000039203 | RGD1566136 | 0 | 0 | 6.726635251 | 3.03E-06 | 0.002187706 |
| ENSRNOG00000061943 | Rn60_7_1214.2 | 0.116044846 | 0 | 6.594327587 | 1.41E-06 | 0.001138846 |
| ENSRNOG00000012067 | Fam111a | 0.067805401 | 0 | 6.468545722 | 1.32E-30 | 8.88E-27 |
| ENSRNOG00000049878 | Sytl2 | 0.219191092 | 0 | 4.984632908 | 3.00E-14 | 5.53E-11 |
| ENSRNOG00000022490 | LOC100361008 | 1.007858866 | 0 | 4.96301805 | 8.02E-31 | 8.12E-27 |
| ENSRNOG00000049491 | RT1-DMb | 0.278876992 | 0 | 4.296689716 | 1.61E-10 | 2.17E-07 |
| ENSRNOG00000002459 | Fbxo40 | 0.020911785 | 0 | 4.246014818 | 5.98E-06 | 0.003903941 |
| ENSRNOG00000017286 | Ephx2 | 0.648425281 | 0 | 4.064497598 | 1.59E-27 | 6.43E-24 |
| ENSRNOG00000055672 | Gpx2 | 2.967941188 | 0 | -2.892930419 | 3.22E-05 | 0.016708588 |
| ENSRNOG00000037607 | LOC100911575 | 185.1100043 | 0 | -2.955941309 | 4.97E-08 | 5.29E-05 |
| ENSRNOG00000012283 | Chrna6 | 12.85501905 | 0 | -3.078948029 | 9.15E-16 | 1.85E-12 |
| ENSRNOG00000019194 | Pitx3 | 7.054565492 | 0 | -3.186673735 | 2.02E-10 | 2.55E-07 |
| ENSRNOG00000027433 | LOC100910708 | 1.871972728 | 0 | -3.202792378 | 8.00E-06 | 0.005060795 |
| ENSRNOG00000048672 | Tmem207 | 0.308526571 | 0 | -3.479338161 | 1.26E-05 | 0.007725743 |
| ENSRNOG00000020410 | Th | 136.842781 | 0 | -3.635146589 | 1.12E-20 | 3.25E-17 |
| ENSRNOG00000012448 | Chrnb3 | 6.133000446 | 0 | -3.758645967 | 1.12E-18 | 2.84E-15 |
| ENSRNOG00000049743 | NEWGENE_620381/GSTM3 | 20.15883853 | 0 | -4.301049538 | 5.29E-27 | 1.78E-23 |
| ENSRNOG00000017302 | Slc6a3 | 116.0051349 | 0 | -6.210375485 | 1.75E-28 | 8.86E-25 |

Note: C = saline control group (n = 3 rats), M = 6-OHDA PD rat model (n = 3 rats).

**Supplementary Table S8.** The top 10 (upregulated and downregulated) DEGs in the Hippo between 6-OHDA PD rat models and saline controls.

| Gene_id | Gene_name | CHippo_FPKM | MHippo_FPKM | Log2FoldChange | Pvalue | Padj |
| --- | --- | --- | --- | --- | --- | --- |
| ENSRNOG00000051360 | RGD1563270 | 0 | 0.95244581 | 7.691940098 | 5.50E-05 | 0.027618155 |
| ENSRNOG00000048232 | Olr1747-ps | 0 | 0.525965799 | 7.048279976 | 2.19E-07 | 0.000337511 |
| ENSRNOG00000031065 | Rn50_20_0045.3 | 0 | 0.500041965 | 6.98711207 | 1.67E-07 | 0.000278209 |
| ENSRNOG00000045695 | NEWGENE_1559832/Rlim/Rnf12 | 0.022419811 | 1.855965806 | 6.288139953 | 5.57E-26 | 3.72E-22 |
| ENSRNOG00000001756 | LOC100911374 | 0.00839392 | 0.749764617 | 6.003238137 | 5.06E-05 | 0.02671512 |
| ENSRNOG00000061943 | Rn60_7_1214.2 | 0.285489409 | 14.98445086 | 5.644034347 | 5.18E-28 | 5.20E-24 |
| ENSRNOG00000012067 | Fam111a | 0.032578373 | 1.669977087 | 5.602809755 | 2.04E-06 | 0.00254184 |
| ENSRNOG00000047000 | Fuom | 0.077071468 | 3.828461607 | 5.522164518 | 4.09E-08 | 8.20E-05 |
| ENSRNOG00000040005 | LOC108348144 | 1.093467631 | 43.71592007 | 5.311427099 | 2.16E-06 | 0.00254184 |
| ENSRNOG00000022490 | LOC100361008 | 0.600457573 | 21.60221629 | 5.156088549 | 2.73E-28 | 5.20E-24 |
| ENSRNOG00000022321 | Hapln3 | 2.815517905 | 0.7995325 | -1.81524758 | 2.66E-05 | 0.016162351 |
| ENSRNOG00000047618 | LOC100910196 | 1.965807911 | 0.336470594 | -2.544498628 | 7.02E-05 | 0.033512259 |
| ENSRNOG00000019477 | Zmynd15 | 0.45994564 | 0.070680301 | -2.661343004 | 6.64E-06 | 0.005327298 |
| ENSRNOG00000020781 | LOC103690005 | 9.396013221 | 1.359074561 | -2.78205921 | 2.57E-06 | 0.002712237 |
| ENSRNOG00000048231 | Tmem235 | 1.414929478 | 0.130532512 | -3.385419524 | 1.54E-05 | 0.009965193 |
| ENSRNOG00000021345 | Timd2 | 0.436810331 | 0.03475937 | -3.584741094 | 1.17E-05 | 0.008077283 |
| ENSRNOG00000027433 | LOC100910708 | 1.496015969 | 0.09121766 | -3.950061944 | 0.000110746 | 0.049340923 |
| ENSRNOG00000019711 | Isoc1 | 5.343512171 | 0.124511982 | -5.388588229 | 9.84E-05 | 0.045857615 |
| ENSRNOG00000020009 | Npas4 | 3.397558717 | 0.037114553 | -6.435727194 | 1.09E-05 | 0.007790477 |
| ENSRNOG00000012575 | Fat2 | 0.228433461 | 0.000780833 | -7.765829808 | 2.18E-05 | 0.013654832 |

Note: C = saline control group (n = 3 rats), M = 6-OHDA PD rat model (n = 3 rats).

**Supplementary Table S9.** The top 15 node genes ranked by degree in the PPI network of striatum.

| Name | Degree | Number of directed edges |
| --- | --- | --- |
| ENSP00000334448(ENSRNOG00000048980/Gng2) | 36 | 36 |
| ENSP00000306245(ENSRNOG00000008015/Fos) | 28 | 28 |
| ENSP00000321106(ENSRNOG00000007374/Tac1) | 25 | 25 |
| ENSP00000352839(ENSRNOG00000015863/Npsr1) | 21 | 21 |
| ENSP00000256010(ENSRNOG00000004179/Nts) | 21 | 21 |
| ENSP00000414303(ENSRNOG00000047466/Bdnf) | 21 | 21 |
| ENSP00000335657(ENSRNOG00000019321/Cck) | 19 | 19 |
| ENSP00000335311(ENSRNOG00000010997/Ednrb) | 19 | 19 |
| ENSP00000324248(ENSRNOG00000008943/Penk) | 19 | 19 |
| ENSP00000276571(ENSRNOG00000012703/Crh) | 17 | 17 |
| ENSP00000293330(ENSRNOG00000018892/Hcrt) | 17 | 17 |
| ENSP00000346017(ENSRNOG00000011130/Calca) | 16 | 16 |
| ENSP00000303325(ENSRNOG00000009372/Tacr3) | 15 | 15 |
| ENSP00000304767(ENSRNOG00000014232/P2ry1) | 14 | 14 |
| ENSP00000334448(ENSRNOG00000048980/Chrm3) | 14 | 14 |
